# Progress in iPSC-Based Modeling of Psychiatric Disorders

**DOI:** 10.3390/ijms20194896

**Published:** 2019-10-02

**Authors:** Anke Hoffmann, Michael Ziller, Dietmar Spengler

**Affiliations:** Department of Translational Research in Psychiatry, Max-Planck Institute of Psychiatry, 80804 Munich, Germany; hoffmann@psych.mpg.de (A.H.); michael_ziller@psych.mpg.de (M.Z.)

**Keywords:** iPSC, schizophrenia, brain organoid, hippocampal circuitry, microglia, cortical interneurons, chimeric mice

## Abstract

Progress in iPSC-based cellular systems provides new insights into human brain development and early neurodevelopmental deviations in psychiatric disorders. Among these, studies on schizophrenia (SCZ) take a prominent role owing to its high heritability and multifarious evidence that it evolves from a genetically induced vulnerability in brain development. Recent iPSC studies on patients with SCZ indicate that functional impairments of neural progenitor cells (NPCs) in monolayer culture extend to brain organoids by disrupting neocorticogenesis in an in vitro model. In addition, the formation of hippocampal circuit-like structures in vitro is impaired in patients with SCZ as is the case for glia development. Intriguingly, chimeric-mice experiments show altered oligodendrocyte and astrocyte development in vivo that highlights the importance of cell–cell interactions in the pathogenesis of early-onset SCZ. Likewise, cortical imbalances in excitatory–inhibitory signaling may result from a cell-autonomous defect in cortical interneuron (cIN) development. Overall, these findings indicate that genetic risk in SCZ impacts neocorticogenesis, hippocampal circuit formation, and the development of distinct glial and neuronal subtypes. In light of this remarkable progress, we discuss current limitations and further steps necessary to harvest the full potential of iPSC-based investigations on psychiatric disorders.

## 1. Introduction

Psychiatric disorders are among the conditions with the largest disease burden [1] and rank fifth globally in causes of disability. Schizophrenia (SCZ) and mood disorders (i.e., major depression (MD) and bipolar disorder (BD)) are associated with high morbidity and mortality. This course reflects ill health as a result of co-morbid cardiovascular or metabolic complications and heightened suicide rates. Consequently, life expectancy is cut by 10 to 15 years in SCZ with no cure available yet [2]. During the course of disease, recurrent episodes become worse and patients undergo incomplete remission; both processes concur with poor therapeutic responses and insidious hospitalization [3]. All of these factors place an enormous burden on patients and their families and contribute to extensive costs (i.e., health care, disability, and lost income).

The intricacy of the human brain, its inaccessibility in life, and a shortage of neuropathological clues to disease mechanism have limited research on psychiatric disorders from the very beginning. This dilemma has somewhat improved with the advent of structural magnetic resonance imaging in which patients with SCZ revealed thinning of prefrontal and temporal cerebral cortices [4]. Though postmortem studies have attributed these changes to reduced synaptic spine density [5,6], which also occurs to some degree in BD [7], these structural abnormalities do not inform on the potential molecular and cellular mechanisms at work. By contrast, well-known pathognomic signs in neurodegeneration (e.g., amyloid plagues in Alzheimer disease) have guided insight into the eventual disease mechanisms. Even worse, changes in brain function such as cognition, mood, and behavior are highly specific to humans and notoriously difficult to model in laboratory animals, in which they need to be induced and maintained experimentally [8]. On top of these concerns, current psychiatric diagnosis relies on historically grown numerous discontinuous categories on disorders [9] that are better conceptualized as quantitative deviations from health. In fact, psychiatric symptoms appear to be rather continuous in nature and thus cross conventional diagnostic boundaries [10].

Notwithstanding these uncertainties, it is well-established that SCZ and BD are highly heritable in twin and family studies [11]. Therefore, risk for psychiatric disorders is thought to be encoded for to a substantial degree in the human genome. Progress in molecular genetics and the advent of high-throughput genotyping techniques have delivered powerful tools to identify, on a genome-wide scale, associations between variations in DNA sequence and particular traits. Current genome-wide association studies (GWAS) have unraveled the complex genetic architecture of psychiatric disorders, in which numerous risk alleles, each of minor effect, incrementally contribute to disease risk, and in which SCZ and BD are closely related genetically (for recent updates, see [12,13]. In aggregate, these studies also support that SCZ and BD evolve from vulnerable brain development [14] and thus strengthen previous epidemiological evidence. The broadly accepted ‘neurodevelopmental hypothesis’ of SCZ [15] postulates that genetic risk factors act in early neurodevelopment, to evoke latent deviations that underpin SCZ onset in adolescence to early adulthood.

Despite remarkable progress in genetics, psychiatric disorders continue to challenge our ability to link genetic associations with molecular and cellular mechanisms that could inform on cognitive, behavioral, and emotional impairments. In fact, current efforts to translate genetic associations into insights on disease mechanisms have been severely delayed by the dearth of appropriate human model systems for dissecting the polygenic disease architecture of psychiatric disorders in the context of living human cell-types from the central nervous system.

Fortunately, this situation has profoundly changed with the transformative discovery [16] that primary cells from various sources (e.g., nuclear blood cells or skin fibroblast) can be converted into a pluripotent state by introducing a cocktail of regulatory factors. Such ‘reprogrammed’ cells, termed human-induced pluripotent stem cells (iPSCs), recapitulate by and large the features of embryonic stem cells (ESCs). Following reprogramming, iPSCs can be differentiated into nearly any cell type including those pertinent to psychiatric disorders. By maintaining the donor’s original genotype, iPSCs offer unique access to the relationship between genotype and genetically encoded cellular phenotypes in vitro, so-called endophenotypes (in psychiatric genetics, the term endophenotype is used to bridge between high-level symptoms (behavior, cognition, emotion) and low-level genetic variability, for example single nucleotide polymorphisms [17]). RNA expression profiling has shown that iPSC-derived neural human cells follow closely the development from early embryogenesis to late fetal periods in vivo and differentiate in vitro into neuronal cells of various stages of maturity [18]. While caution is necessary to extrapolate from iPSC-derived cell stages to those in adolescents and adults, they offer unprecedented access to the role of genetic risk factors in early human neurodevelopment and to the delineation of critical time windows through which these factors act on molecular and cellular phenotypes.

Following pioneering work by Brennand et al. [19], the number of iPSC-based case/control studies on SCZ and BD has increased steadily over the last years (reviewed in [20,21,22]). These studies measured in neuronal cells from patients with SCZ (i) reduced neuronal connectivity, (ii) attenuated activity-dependent transcription, (iii) impaired mitochondrial function and increased oxidative stress, (iv) delayed NPC differentiation and neuronal maturation, and (v) altered miRNA expression. A similar picture has emerged for BD with deregulation of different signaling pathways, mitochondrial function, miRNA expression, and NPC proliferation. In distinction to SCZ, iPSC-derived neurons from patients with BD also showed a hyperexcitable phenotype that normalized in response to lithium treatment in vitro.

Overall, these studies present remarkable progress in the field, though they also indicate the need for further improvements. Therefore, we will discuss in this review recent advancements on iPSC studies in psychiatric disorders that seek to address present challenges, both in terms of experimental design and new techniques. Concluding, we also consider present hurdles to overcome for harvesting the full potential of iPSC-based studies in psychiatric disorders.

## 2. Reconstructing Human Brain Development and Circuitries In Vitro

Our insight into the structural, cellular, and circuit-level deviations that manifest in early brain development in patients with SCZ and BD is at present very limited. Recent progress in three-dimensional (3D) culture models holds great potential for producing brain-like structures (hereafter referred to as ‘brain organoids’ or ’spheroids’) from patient-specific iPSCs. Thereby, different cell types are thought to recapitulate anatomically relevant spatial organization, in which cellular connections might more closely track those observed in vivo than in conventional 2D-systems [23]. In fact, broad features of the developing brain, such as radial organization of cell types around ventricles, are retained in brain organoids and spheroids.

**Figure 1:**
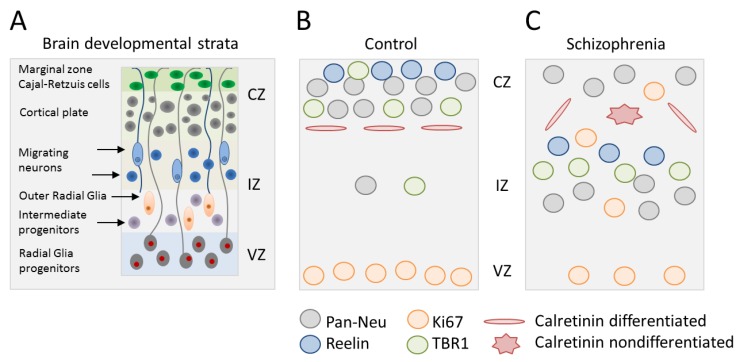
Disrupted neocorticogenesis in forebrain organoids from patients with schizophrenia (SCZ). (**A**) Schematic of neocorticogenesis with different developmental strata and key cell types indicated. Germinal zones consist of the ventricular, intermediate, and cortical zone (VZ, IZ, and CZ). Radial glia cells undergo mitosis at the ventricular surface and hold contact with the basal lamina via their basal process. Intermediate progenitors and outer radial glia cells divide at an abventricular location without and with ventricular contact, respectively. Newborn neurons migrate along basal processes to reach their final destination. (**B**,**C**) Synopsis of results from forebrain organoids from patients with SCZ relative to healthy controls: (i) Enhanced proliferation of neural progenitor cells (NPCs) (Ki67-positive), which migrate outside the VZ into the IZ and CZ, (ii) reduced neocortical expression of reelin that directs cortico-petal migration, (iii) decreased cortical accumulation of TBR1-positive neurons leads to less cortical neuron formation, (iv) attenuated cortical neuron development in favor of subcortical neuron development, and (v) less calretinin positive interneurons, which form horizontal processes in order to connect cortical column. Adapted from [24].

In a recent study, Stachowiak et al. [24] investigated brain organoids generated from iPSCs of three patients with SCZ and of three unaffected family member. These iPSCs were originally established by Brennand et al. [19] and have been extensively characterized since then (reviewed in [20,22]). A 3D-protocol of directed early patterning was used to establish forebrain-like regions (Figure 1A), which exhibited no gross differences between controls versus patients with SCZ (Figure 1B,C). However, microscopic analysis showed significant cellular misalignments: From week 2 onward, control organoids contained, in the ventricular zone (VZ), 2–3 layers of proliferating NPCs, in the intermediate zone (IZ) few proliferating NPCs, and in the cortical zone (CZ) hardly any proliferating NPCs (Figure 1B). By contrast, forebrain organoids from patients with SCZ showed only one layer of proliferating NPCs in the VZ with no palisades surrounding the VZ lumens. Instead, proliferating NPCs were dispersed in both the IZ and CZ (Figure 1C). Expectedly, differentiating neurons resided by two weeks in the CZ of organoids from controls, but were still retained in the IZ and VZ of organoids from patients with SCZ. Cortical regions of organoids from patients with SCZ also showed a reduction in T-Box Brain 1-positive neurons (TBR1, a canonical factor for neurodevelopment), and a loss in directionality and connectivity of calretinin-positive interneurons.

Overall, this study on forebrain organoids firstly showed aberrant subcortical neurogenesis in vitro in patients with SCZ: (i) Proliferative NPCs were retained in the IZ and gave rise to atypically placed deep subcortical neurons, (ii) TBR1-positive pioneer neurons stayed behind in the IZ and fell short to immigrate into upper cortical layers, and (iii) calretinin-positive interneurons exhibited random directionality of neurites, suggesting perturbed intercortical connectivity. While these results are consistent with those from previous 2D-models on impaired migration, differentiation, and connectivity of iPSC-derived NPCs and neuronal cells from patients with SCZ [19,25,26], they take us an important step further by utilizing a reductionist model of human brain development. This allows insight into the cellular and structural consequences of altered monolayer culture regarding subcortical neurogenesis in vitro, and by extrapolation, early brain development in patients with SCZ. However, caution is necessary given the small size of the present study [24] that calls for additional studies on familiar and idiopathic SCZ.

Despite the raising interest in iPSC-based modeling of psychiatric disorders, a suitable system to investigate well-defined human neuronal circuitries has been lacking until recently. In fact, almost all current studies [20,22] have sought to establish neuronal monolayer cultures consisting of fairly homogenous populations of a defined subtype (e.g., excitatory cortical neurons) lacking the complex network activity seen in vivo. The hippocampus is of particular relevance in this respect since its individual cell types, their gene-expression profiles, and their organization in regular circuits are well-known. For example, presynaptic dentate gyrus (DG) axons connect via the mossy fiber (MF) pathway to postsynaptic CA3 neurons. This circuit undergoes constant modifications in response to developmental and environmental signals and thus underpins neuronal plasticity in health and disease. Along this line, current neuroimaging studies agree that neuronal connectivity in prefrontal cortex, among other cortical brain regions, is altered in SCZ and could contribute to impaired hippocampus-dependent cognitive functions [27].

To model hippocampal circuitry in psychiatric disorders, Sarkar et al. [28] recently refined a directed differentiation protocol for hippocampus-patterned NPCs that closely mimics in vivo development [26]. Briefly, patterned NPCs were either differentiated for only three weeks in the presence of a low dose of wingless to obtain CA3-like neurons or with a high dose of wingless over the entire differentiation period to obtain DG-like neurons.

CA3-like pyramidal neurons consisted mainly of glutamatergic cells (> 90%) with gene expression profiles closely matching the CA3 and the broader CA3 subfields (latter comprises both CA2 and CA3) as evidenced by single-cell sequencing. By contrast, genes specific to DG neurons were weakly expressed in six-week-old hippocampal cultures. In support of their mature stage, CA3-like, but not DG-like neurons, were electrically active (Na^+^ and K^+^ currents, spontaneous and evoked action potentials, and spontaneous excitatory postsynaptic currents) and developed dense electrically active connections (measured by spreading of rabies virus [19] and multiple-electrode arrays). Note, too, that this behavior of iPSC-derived human CA3/DG3-like neurons matched the one of mouse CA3/DG3 neurons differentiated for four weeks in vitro and thus seemed to reflect the differential maturation competence of hippocampal neuronal subpopulations. Even though, co-culture of human CA3-like and DG-like neurons promoted the development of an electrically active DG-CA3 circuit in a cell-autonomous manner (i.e., no exogenous axon guiding signals or afferent neuronal activity were required) indicating that CA3-like neurons enhanced the maturation of DG3-like neurons.

Having established a system to assess hippocampal DG-CA3 circuit formation in vitro, Sarkar et al. moved on to compare iPSC samples from controls versus patients with familial SCZ [19]. Intriguingly, spontaneous spike and network burst were less frequent in iPSC-derived DG-CA3 co-cultures from patients with SCZ relative to controls at six weeks of in vitro differentiation. Since case/control co-cultures contained comparable few inhibitory GABAergic interneurons (< 5%), these differences seemed to reflect differences in their excitatory, rather than their inhibitory, activity. When either CA3 population was tested on its own, neural network activity was attenuated in CA3 neurons derived from patients with SCZ relative to controls. However, CA3 neurons did not differ between cases and controls at earlier stages of differentiation in vitro (week 1 to 5) in terms of electrical activity. Hence, these differences seemed to evolve during the course of later neuronal maturation.

Taken together, this study throws light on the question of how deficits in the maturation of hippocampal CA3 neurons can impact DG-CA3 circuitry, and by implication, cognitive processes in patients with SCZ. It is important to note that previous work on iPSC-derived neurons has indicated impaired synaptic maturation, neuronal connectivity, and aberrant neurotransmitter secretion in patients with SCZ when compared to controls [19,26,29]. Still, the question of how cellular alterations in ‘one cell type only’ systems could translate in altered circuit function relevant to cognition and behavior has remained poorly explored as of yet. Here, Sarkar et al. make an important step forward to meet this challenge by establishing a cellular system that could serve as a blueprint for further in vitro studies on hippocampal pathologies in psychiatric disorders.

Advanced iPSC culture models would not only promote investigation of circuit-level abnormalities, but also of altered cell–cell interactions in psychiatric disorders. For example, altered neuron–oligodendrocyte interactions are thought to contribute to abnormal myelination [30], and altered neuron–microglia interactions have been implicated in excessive pruning activity in SCZ [31].

As the brain develops, it produces an excess of synapses during early stages. Upon maturation, neural circuitries are reshaped by environmental information (i.e., sensory, cognitive, and social) that triggers the elimination of redundant synapses, while strengthening the remaining ones. This process, called ‘synaptic pruning’, occurs also alongside the refinement of cognitive functions in late adolescence and early adulthood [32] when SCZ typically manifests. Accordingly, Irwin Feinberg [33] hypothesized that enhanced or irregular pruning activity could contribute to behavioral and cognitive impairments in patients with SCZ. Until recently, cellular in vitro models to study the molecular and cellular processes underpinning such aberrant pruning were rather unknown.

To fill this gap, Sellgren et al. [34] set up an iPSC-based in vitro model of microglia-mediated engulfment of neuronal synapses. In a preliminary study, the researchers converted (i.e., ‘reprogrammed’) human peripheral blood mononuclear cells into microglia-like cells by treatment with a cytokine cocktail [35]. Notably, induced human microglia-like cells (hiMGs) displayed a ramified morphology mimicking resting state microglia and closely recapitulated in vivo marker and gene expression profiles. Furthermore, hiMGs engulfed iPSC-derived neuronal synaptosomes and NPCs in a complement-dependent manner in vitro.

**Figure 2:**
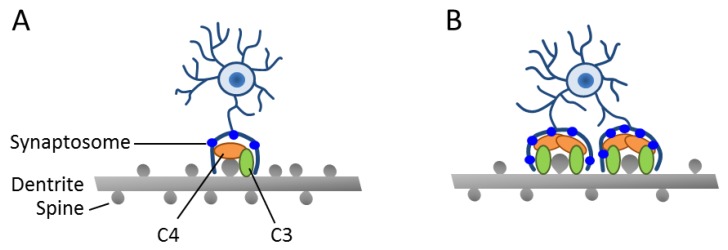
Enhanced synapse engulfment by human-induced microglia cells (hiMGs) in a human-induced pluripotent stem cells (iPSC)-derived cellular in vitro model from patients with SCZ. (**A**,**B**) Schematic depicts a co-culture of hiMGs and iPSC-derived neurons from a healthy donor (**A**) or a patient with SCZ (**B**). Moderate dendritic pruning with a slight decrease in spine density is detected in the healthy donor. By contrast, excessive pruning takes place in the patient-derived cellular model and results in decreased spine density. This pruning phenotype is associated with an increased deposition of C3 complement in iPSC-derived neural cultures from a patient with SCZ. Adapted from [36], license number: 4622480163536.

To assess pruning activity in SCZ, Sellgren et al. [34] moved on to produce hiMGs from case and control subjects. Quantification of hiMG-dependent synaptosomal phagocytosis or of co-cultured neurons’ spine density revealed excessive pruning activity in patient-derived cellular models, which involved both microglial and neural factors (Figure 2).

In the latter respect, a previous study [37] has shown that genetic risk for SCZ relates in part to the complement component 4 (C4) genes that reside within the histocompatibility complex on chromosome 6, a region that presents, by far, the strongest risk association. The complement system is thought to tag pathogens and cell debris, and thus enhance clearance by phagocytic cells. Beyond the immune system, the same mechanism has been co-opted by the nervous system in synaptic pruning [38]. Thereby, C4 induces C3 activation, leading to covalent binding onto its targets followed by subsequent phagocytic engulfment. In fact, C3 deposition strongly correlated with the presence of the C4 risk allele in neural cultures generated from iPSC’s of patients with SCZ and led to increased synaptosom engulfment in vitro (Figure 2).

Intriguingly, treatment with minocycline, a broad-spectrum tetracycline antibiotic, revealed a dose-dependent inhibition of synaptosom engulfment in vitro at clinically relevant doses suggesting a potential treatment of individuals at risk. Consistent with this possibility, analysis of large medical health records with a focus on individuals between the ages of 10 and 18, and with at least one electronically prescribed tetracycline, indicated that treatment at a minimum of 90 days was associated with a significant reduction in the risk for incipient SCZ [34].

Taken together, Sellgren et al. showed that increased hiMG-mediated synaptosomal phagocytosis in vitro are associated with C4 risk variants [37] and may be prevented by minocycline treatment in incipient SCZ. Despite these exciting findings, we would like to caution that hiMGCs trace gene expression profiles of human ex vivo microglia only to some extent and that the cellular model systems do not recapitulate experience-dependent neuronal activity regulating synaptic pruning in vivo. Apart from neuronal cells, the role of C4, or other still unknown risk variants, in human microglia also requires further studies to round off our understanding of hyperactive pruning in patients with SCZ. Even then, Sellgren et al. strengthen current evidence for enhanced synapse elimination in SCZ and provide mechanistic insight into how genetic risk variants could bridge to altered brain structure in incipient stages of disease.

## 3. In Vivo Studies of Patient-Specific iPSCs

When considering implications from iPSC-based modeling of psychiatric disorders, it is important to stay aware that genes do not encode for psychopathology. As noted before, chromosomal regions and genes associated with psychiatric disorders are numerous, with each genetic variant conferring only subtle changes at the molecular and cellular level. Most of these alterations are predicted to occur within neurons [39,40], thus affecting the formation of micro- and macro-circuits during early brain development and beyond, most likely in a tissue-specific manner [41]. Behavior arises from neuronal circuits processing environmental information, and aberrant circuit physiology is thought to contribute to psychiatric disorders. For example, imbalances in excitatory–inhibitory signaling (Zikopoulos and Barbas, 2013) (see below) or cell–cell interactions [31] (see above) can alter circuit functions, and by implication, impact psychiatric disorders. In the field of psychiatry, iPSC-based case/control studies need to meet the requirement that molecular and cellular differences detected in vitro are relevant to behavioral changes in vivo. Therefore, we refer next to iPSC-based studies that sought to tackle this question to some extent by combining transplantation and behavioral studies in mice.

A number of imaging studies on psychiatric patients have suggested disrupted synaptic connectivity in SCZ, preferentially in the prefrontal cortex [42,43]. Emerging evidence indicates that such lesions could result from dysfunctional oligodendrocyte precursors and/or oligodendrocytes. These cells carry out myelination of axons in white matter to boost neuron conduction. At present, it is unknown whether potential deficits in oligodendrocyte function present a primary or a secondary cause of altered neuronal and synaptic functions. In any case, these abnormalities are already detectable in infants at high risk for SCZ [44] and children with early onset of SCZ (COS) [45]. COS is a rare disorder (1 in 10,000–30,000) with greater neurodevelopmental deviance early in life, yet clinically and neurobiological continuous with adult onset SCZ. Similar to other fields of early-onset disease, COS comes with a greater genetic burden [46] suggesting that it could be particular informative to iPSC-disease modeling of early neurodevelopmental perturbations [22] and of disease traits that unfold more subtly in adult-onset patients.

**Figure 3:**
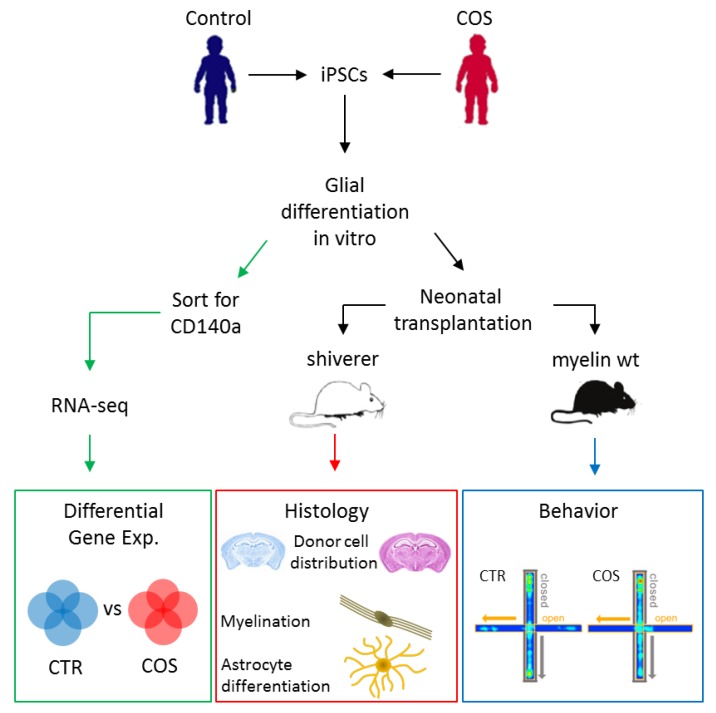
Experimental flowchart for the analysis of control- and children with early onset of SCZ (COS)-derived iPSCs. Quality-controlled iPSCs were differentiated in vitro into human glial precursor cells (hGPCs) that were subsequently transplanted in myelin-deficient shiverer or myelin-wildtype neonate mice. Histological analysis (middle) was applied to assess the distribution of donor cells in the recipient host brain, the extent of myelination, and the development and differentiation of astrocytes. In parallel, control or case hGPCs-engrafted myelin-wild-type mice (right) were exposed to a battery of behavioral tests to investigate phenotypes thought to mimic those from patients with COS/SCZ. Additionally, in vitro differentiated hGPCs were enriched for the lineage-specific marker CD140a by FACS and subsequently used in RNA-sequencing (left) to determine differentially expressed genes between healthy individuals and patients with COS. Adapted from [47]., license number: 4622481473507.

Against this background, Windrem et al. [47] established iPSCs from a cohort of five patients with COS and four age-matched healthy individuals. Following quality control, iPSCs were differentiated into human glial precursor cells (hGPCs). These cells were engrafted into neonatal immunodeficient shiverer mice that show congenital hypomyelination owing to the absence of myelin basic protein (MBP) (Figure 3). During the course of maturation, control transplanted hGPCs developed into both astrocytes and myelinogenic oligodendrocytes and gave rise to mostly humanized forebrain white matter. Thereby, hGPCs generated from controls consistently increased in numbers through the white matter before invading the cortical gray matter. By contrast, hGPCs derived from patients with COS entered the white matter early, traversed without pausing, and produced less donor hGPCs. In parallel to premature cortical entry, hGPCs derived from patients with COS were impaired in oligodendrocyte differentiation and caused a deficit in central myelogenesis. Moreover, astrocytic differentiation was impaired as well, with cells showing fewer primary processes, proximal branching, and coherent domain structures.

RNA-sequencing of in vitro differentiated hGPCs from patients with COS (Figure 3) showed, relative to controls, downregulation of genes driving early oligodendroglial and astroglial lineage progression. Among these genes were key transcription factors (e.g., *OLIG1*, *OLIG2*, *SOX10,* and *ZNF488*) and early-stage regulated proteins from myelination (e.g., *GPR17*, *UGT8*, *OMG,* and *FA2H*). Moreover, a group of disease-linked ion channels and genes regulating synapse development and function were downregulated in hGPCs from patients with COS. Together, these data indicate that reduced myelination in shiverer brains transplanted with hGPCs from patients with COS results from perturbed oligodendrocyte and astrocyte differentiation.

Any impairment in the differentiation of astrocytes could contribute likewise to the pathogenesis of SCZ given their critical role in synaptic development and function [48]. To test this hypothesis, Windrem et al. [47] moved on to assess the behavior of immunodeficient, but otherwise normally myelinated, neonatally engrafted mice (Figure 3). Interestingly, recipients of hGPCs from patients with COS showed, relative to those engrafted with hGPCs from controls, significantly diminished auditory prepulse inhibition. This measure is a proxy to sensorimotor gating whose decrease may predict certain phenotypes in SCZ. Engrafted mice were further exposed to a battery of cognitive and socialization tests (elevated plus maze, an anxiety test; three-chamber social challenge; novel object recognitions, an executive memory test; and sucrose preference, an anhedonia test) and to an analysis of sleep and diurnal activity patterns. Collectively, glial chimerization with cells from patients with COS was associated, in engrafted recipients, with increased anxiety and fear, and with concurrent deficits in socialization, cognition, and sleep patterning, relative to those counterparts engrafted with cells from controls. All of these behavioral symptoms are frequently observed in patients with COS and SCZ.

Taken together, this study suggests that cell-autonomous deficits in glial development could contribute substantially to COS pathology. Beyond impaired myelogenesis upon engraftment of patient GPCs, defects in astrocytic maturation led to substantial cognitive and behavioral impairments in recipient mice relative to those counterparts transplanted with GPCs from healthy controls. Although differences in cell viability between case/control samples upon transplantation remained untested [47], a salient explanation for this phenotype is altered circuit formation and synapse development in mice transplanted with GPCs from patients with COS. In this respect, an earlier report [49] showed that humanized chimeric mice exhibited enhanced long-term potentiation and learning capacity when compared to allografted or untransplanted controls and thus suggest that altered circuit physiology contributes to the differential effects in COS versus control GPC transplanted mice.

As a final remark we note that Liu et al. [50] recently capitalized on these findings to provide further insight into impaired astrocytic differentiation in patients with COS. Pathway analysis of previous RNA-seq data revealed enhanced BMP (bone morphogenetic protein) signaling in GPCs from patients with COS relative to controls. This event, in turn, stimulated overexpression of negative feedback regulators in BMP signaling and thus blocked astrocytic differentiation in vitro. Consistent with this finding, knockdown of SMAD, a downstream effector of BMP signaling, restored astrocytic differentiation of GPCs from patients with COS. In addition, a small number of astrocytes from patients with COS that accomplished differentiation were functionally impaired owing to downregulation of a broad group of genes encoding for potassium channels. Most of them shared, in their upstream regulatory regions, DNA-bindings sites for the transcriptional repressor REST, which is, to some extent, under the control of BMP/SMAD-signaling and was upregulated in GPCs from patients with COS.

Collectively, these findings suggest that dysregulated BMP signaling restricts astrocytic differentiation in vitro of GPCs from patients with COS and impairs functional maturation owing to REST-mediated downregulation of potassium channel genes. The latter event could lead to enhanced interstitial K^+^ concentrations, reduced neuronal firing thresholds, and consequently accentuate network desynchronization. In this situation, agents promoting glial K^+^ uptake may present an opportunity toward relieving glial maturation defects in COS/SCZ and further studies are warranted to explore this option in chimeric-hGPC mice.

Imbalances in excitatory–inhibitory signaling are widely viewed as a mechanism for cortical dysfunction in SCZ owing to an enhanced excitatory glutamatergic and/or a diminished inhibitory GABAergic tone (Zikopoulos and Barbas, 2013). In support of this hypothesis, integration of postmortem brain transcriptomics of major psychiatric disorders [51] with single-cell expression datasets suggests that SCZ risk loci primarily comprise candidate genes expressed in cortical pyramidal excitatory neurons and a subset of GABAergic interneurons [40]. Cortical inhibitory interneurons (cINs) belong to the cell types most consistently affected in postmortem studies [52], particularly those of the parvalbumin- (PV^+^) or somatostatin- (SST^+^) positive subtype, which originate from the medial ganglionic eminence. In vivo, altered GABAergic signaling seems to disrupt cortical γ-oscillations in patients with SCZ and thus contribute to cognitive impairments [53]. Cognitive deficits typically emerge prior to the onset of psychotic symptoms and frequently present in children with developmental delay or at high risk for SCZ. Together, these findings indicate that the pathogenetic process leading to altered function of prefrontal GABAergic interneurons in SCZ could root at early neurodevelopmental deviations.

**Figure 4:**
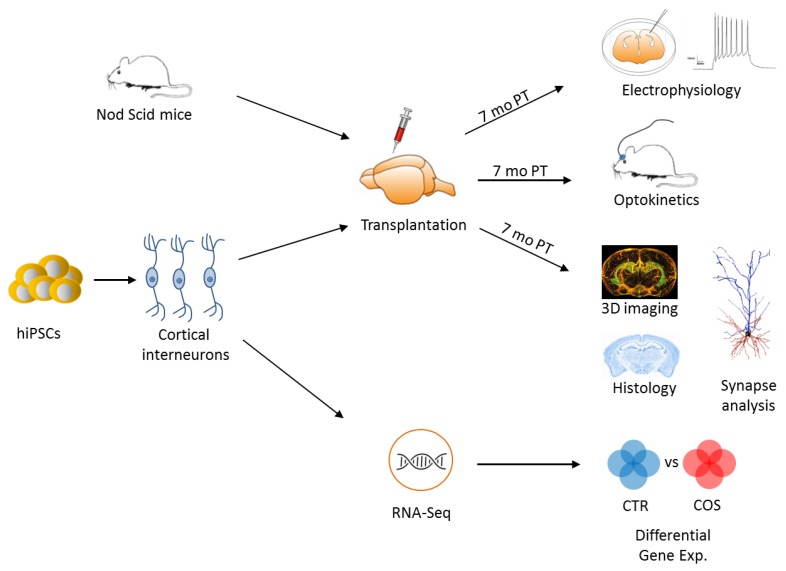
Experimental flowchart for the study of iPSC-derived cortical interneurons. Quality-controlled iPSCs from healthy donors and patients with SCZ and were differentiated in vitro into cortical interneurons (cINs), which were subsequently transplanted in the cortex of adult mice (immune-compromised Nod Scid mice). Seven months post-transplantation (PT), electrophysiological and optogenetic studies were performed in vivo and in vitro to assess the properties of transplanted cINs from controls and cases and to compare them to those of naïve mice cINs. Additional experiments involved 3D imaging and histological analysis of transplanted human cINs. In parallel, in vitro differentiated cINs were RNA-sequenced and analyzed for differences in gene expression between healthy controls and patients with SCZ. Adapted from [54], license number: 4634820039637.

To test this hypothesis, Shao et al. [54] generated iPSCs by footprint-free RNA-mediated reprogramming from each of 14 healthy controls and patients with SCZ. Patients were selected based on prior clozapine treatment, a second-line treatment for severe SCZ. Quality controlled iPSCs were differentiated in cINs and then transplanted into the cortex of immunodeficient male and female mice at an age of 5–7 weeks (Figure 4).

Electrophysiological and optogenetic studies were conducted in vivo and in vitro at seven weeks post transplantation. In a first set of experiments, Shao et al. showed that cINs derived from healthy controls and patients with SCZ developed into cortical neurons closely recapitulating the features of endogenous interneurons, and that both groups expressed functional postsynaptic structures responding to host glutamatergic neurons. Similarly, both groups expressed presynaptic structures for the release of GABA and the inhibition of host cortical neurons. In essence, cINs from both controls and cases undistinguishably developed into authentic and fully functional interneurons in recipient mice.

RNA-seq analysis of in vitro differentiated cINs from controls and cases (Figure 4) showed global gene expression patterns with only few genes differing between the diagnostic groups. These included a subgroup of protocadherin alpha (PCDHA) genes (*PCDHA2, PCDHA3, PCDHA6,* and *PCDHA8*), where downregulation was specific to cINs from cases, but undetectable in glutamatergic-fated neurons derived from iPSCs of either group.

To assess the impact of *PCDHA* downregulation on early neurodevelopment in vivo, Shao et al. [54] turned to *Pcdha*-knockout mice that lacked all *Pcdha* family members. Histological analysis of prefrontal cINs showed reduced formation and arborization of inhibitory synapses, whereas excitatory synapses were unaffected. Consistent with this finding, a previous report [55] had detected deficits in projection and connections in different brain regions, which were associated with deficits in contextual learning and working memory. In the current study, *Pcdha*-knockout mice exhibited additional deficits in prepulse inhibition. Since all of these behavioral alterations are thought to mimic clinical phenotypes in SCZ, they prompt the question whether *Pcdha*-knockout mice matched cINs phenotypes from patients with SCZ in vitro and in vivo, and in postmortem human brain. In fact, somatic neurite, and total branch numbers, as well as total neurite lengths were significantly reduced in vitro in iPSC-derived cINs from patients with SCZ relative to controls. Gain and loss of function experiments further revealed that the deficit in cINs from patients with SCZ could be rescued by treatment in vitro with a PKC inhibitor, or conversely, be induced by knockdown of protocadherin expression in cINs from controls. While cINs fate decision and excitatory synapse formation in vivo did not differ between control- and case-engrafted mice, cINs from cases showed a significant deficit in inhibitory synapse formation relative to controls, thus strengthening the results from in vitro culture. Taken together, these findings indicate that iPSC-derived cINs from patients with SCZ form less inhibitory synapses in the absence of other potential disease-associated alterations in cortical circuitry, an outcome pointing to a cell-autonomous defect. Interestingly, these early developmental cINs phenotypes were recapitulated in a SCZ/control study on postmortem brains: In distinction to early developmental stages in vitro and in vivo, postmortem cINs from patients additionally showed a significant reduction in excitatory synapse formation. Therefore, conditions in vitro would be either not permissive to downregulation of excitatory synapses or this event could reflect a secondary cause in vivo. In light of the small-sized (*n* = 8 for each condition) postmortem brain collection [54], further studies are needed to support these results.

Overall, Shao et al. shed new light on the excitatory–inhibitory imbalance hypothesis in SCZ. Integrated analysis of iPSC-derived cINs from patients with SCZ demonstrated deficits in inhibitory synapse function in models of early brain development in vitro and in vivo, and in postmortem adult human brain. This deficit manifests in a cell-autonomous fashion in vitro, independent of the circuit environment during early brain development in patients with SCZ. At the molecular level, this phenotype resulted, at least in part, from downregulation of *PCDHA* genes. In a complementary approach, *Pcdha*-knockout mice represented cognitive and behavioral deficits mimicking clinical phenotypes from patients with SCZ. While different lines of evidence from this study converge on an early cell-autonomous defect in cINs, less insight has been gained on the precise genetic and molecular mechanisms involved. Particularly, the potential contribution of current risk loci in SCZ to *PCDHA2* regulation remained unsettled owing insufficient sample size. Also, how changes in *PCDHA2* relate to other differentially expressed genes between cINs from controls and patients with SCZ needs to be clarified, though these seemed to be only few. Current progress in genome editing techniques [56] allows dissecting cause–effect relationships in gene regulation and could guide further experiments on causative molecular mechanisms disrupting early cINs development.

## 4. Discussion and Outlook

Present iPSC-based disease modeling has made significant progress in tracing human brain development and early neurodevelopmental deviations in psychiatric disorders. While studies on SCZ are leading, we expect other fields of psychiatry (e.g., BD and MD) to follow in time as the field of iPSC-based disease modeling grows and matures. To date, iPSC studies on patients with SCZ suggest that distinct impairments in NPC proliferation, migration, and differentiation in 2D-culture extend to 3D-brain organoids and disrupt neocorticogenesis in vitro. Likewise, the formation of circuit-like CA3/DG hippocampal structures in vitro is impaired in patients with SCZ, pointing to a possible cause of impaired cognitive and behavioral functions in vivo. Beyond neurons, emerging evidence points to perturbed glia development in patients with SCZ. Elegant chimeric-mice studies support altered oligodendrocyte and astrocyte development in vivo in recipients engrafted with GPCs from patients with COS. These alterations are associated with behavioral and emotional impairments mimicking those of patients. As astrocytes guide neuronal maturation, this study too highlights the significance of cell–cell interactions in the pathogenesis of SCZ. Along this line, cortical imbalances in excitatory–inhibitory signaling may result from a cell-autonomous defect in cINs development that originates, to some extent, from the downregulation of *PCDHA* genes. This gene family regulates cell–cell adhesion in development and beyond, and *Pcdha*-knockout mice exhibited behavioral impairments reminiscent of clinical symptoms in patients with SCZ. In aggregate, these studies indicate that genetic risk in SCZ impacts neocorticogenesis, hippocampal circuit formation, and the development of distinct glial and neuronal subtypes. In light of these remarkable findings, we next discuss potential caveats and further steps to be taken in the field of iPSC-based modeling of psychiatric disorders.

### 4.1. Beyond 2D-Cell Culture

Progress on human iPSC-derived cellular models has stimulated the generation of 3D-brain organoids for investigating early brain development in vitro, and by implication, neurodevelopment-related disorders. All of these iPSC-derived brain organoids require external patterning cues to attain control over specific brain regions and types of cells generated, and importantly, over reproducibility. While brain organoids produce a vast array of cells resembling in vivo counterparts, they exhibit high variability that could confound the analysis of case/control studies [57]. In this regard, Stachowiak et al. [24] relied on iPSC lines that were well-characterized and known to show distinct differences between healthy controls and patients with SCZ in 2D-culture systems. Organoid-to-organoid variability has also prompted concerns about the consistency of developmental processes outside the context of human embryogenesis. Zooming in on an organoid model of dorsal forebrain, Velaco et al. [58] recently showed that patterned organoids contain a nearly indistinguishable repertoire of cell types tracing developmental trajectories with similar variability to those of individual human brains. While this outcome may open the door to case/control studies on patterned brain organoids, a number of unresolved issues remains. Above all, brain organoids recapitulate only in part the cellular diversity and circuit functionality of various brain regions from living beings. This poses a particular challenge to organoid models that strive to generate brain regions containing cells from different regional lineages. For instance, the germinal zones of the dorsal and ventral telencephalon give rise to the excitatory and inhibitory neurons, respectively, of the cerebral cortex. On the contrary, dorsalizing patterning cues promote the formation of an ‘all excitatory’ brain organoid, in which inhibitory interneurons are barely present. Alternatively, ‘brain assembloids’ [59] could be used to model excitatory–inhibitory imbalances presumably involved in altered network physiology in SCZ and other psychiatric disorders. For this purpose, multiple cell lineages are combined in 3D to mimic interactions between various brain regions in vitro, thus enabling deeper insight into the assembly of neural circuits and the formation of complex cell–cell interactions.

Another concern about brain organoids is the inadequate oxygen and nutrient supply owing the absence of vascularization, which restricts progressively maturation. To promote vascularization, growth factors can be supplemented to nascent organoids or neural organoids can be transplanted into the brains of adult mice [60]. Engrafted brain organoids showed not only significantly improved survival, but also progressive neuronal differentiation and maturation (see below). However, such experiments are tedious and require advanced skills to produce and analyze transplants, making it challenging to carry out more extensive investigations on multiple samples from statistically significantly empowered case/control studies (for a discussion on estimated size numbers for iPSC-based studies, interested readers are referred to [20,61]).

Alternatively, a practicable fully in vitro system is needed to recapitulate key features in psychiatric disorders as well as in other brain diseases. In this regard, Park et al. [62] recently introduced a microfluid device in which human stem-cell derived neurons and astrocytes were grown in a 3D-culture matrix with microglia added at later time points. Moving on to patient-specific iPSCs from individuals with Alzheimer’s disease (AD), the researchers found that under the conditions of this triculture system, neurons developed signs of AD pathology in vitro due to microglia-mediated inflammatory processes. By now, such composite cellular in vitro systems appear more suitable for in-depth analysis of preselected iPSC samples from psychiatric case/control studies than for primary screens, both in terms of required technical skills and costs. Even though, advances in vitro 3D-cell culture system can, and will, significantly contribute to unlock the complex biology of psychiatric disorders in the long term.

### 4.2. Building Neural Circuits In Vitro

Ideally, any in vitro cell culture model would capture those abnormalities in patients that arise from multiple cell types, their intricate interactions, and the physiological activity of both local and long-distance neural networks. Presently, we have still to reach out for this goal: Early stages of NPC development, neurogenesis, and differentiation typically prevail in current in vitro models, while rather few touch on initial stages of neural-network formation. In any case, cellular traits characteristic of more mature stages such as postnatal myelination remain, so far, largely elusive (see below). Notwithstanding these reservations, the recent generation of hippocampal circuit-like structures in vitro by Sarkar et al. [28] brings us a step closer to this goal. Note, too, that both patterns of hippocampal gene expression in vitro and that of DG/CA3 circuit formation and functionality in vitro recapitulated those from in vivo in human and mice, respectively. Clearly, a major issue in modeling psychiatric disorders is to produce authentic cell types robustly and to faithfully re-create cell–cell interactions and circuit connectivity of the living brain. This is the litmus test of iPSC-based modeling, if we are to believe that differences between healthy controls and patients with SCZ in hippocampal circuit-like functions in vitro extend to the human brain and thus inform on the pathology involved in cognitive and behavioral impairments. While brain organoids display neural network activity in vitro [57] and in transplanted mice [60], it is, at present, incompletely understood to what degree circuits engaged feature structural and physiological properties of the fetal and postnatal brain. Once this issue has been resolved, neural network formation and activity in brain organoids may provide higher-order information on aberrant circuit physiology in psychiatric disorders.

### 4.3. Transplantation of iPSC Derived Cells

IPSC-derived cells from case/control studies can be transplanted into neonate or adult rodent brains to study their properties in a more ‘naturalistic’ environment that is presumably more permissive than in vitro conditions to disease processes involving migration, connectivity, circuit integration, and advanced morphological and electrophysiological maturation. In addition, this approach can be used to identify deficits in distinct behavioral domains in recipient hosts that are relevant to psychopathology, though it does not allow for recapitulating systems-level functionality underlying behavioral, cognitive, and emotional impairments in patients with psychiatric disorders.

While clinical symptoms in major psychiatric disorders suggest disintegration of neural circuits, we would like to point out that neurons do not operate outside the world of other cell types present in the living brain. Healthy brain function reflects the intricate balance of interactions between neurons, astrocytes, microglia, and vascular cells, which become compromised during disease. Along this line, Windrem et al. [47] recently investigated a role of glia in early neurodevelopment by transplanting iPSC-derived GPCs from healthy controls and patients with COS in neonate mice brains. Interestingly, findings from chimerized brains suggested a role for oligodendrocytes and astrocytes in the development of COS with recipient mice exhibiting behavioral and cognitive phenotypes mimicking those from patients with SCZ. Although COS is clinically and neurobiologically continuous with adult onset SCZ, it is associated with a higher genetic burden, particularly in the form of large chromosomal abnormalities and copy number variations (discussed in [22]). The question arises as to what degree the findings of Windrem et al. can be generalized to idiopathic SCZ. In this regard, Narla et al. [63] have recently found that iPSC-derived neuronal committed progenitor cells from patients with idiopathic SCZ showed an upregulation of genes relevant to differentiation and maturation of neurons and axonal guidance, whereas genes relevant to glial differentiation and myelination were downregulated. In-depth analysis of selected pathways suggested a concerted enhancement of pro-neuronal developmental mechanisms at the expense of pro-glial/anti-neuronal signaling. Therefore, it would be interesting to test to what degree these in vitro findings on idiopathic SCZ translate into in vivo phenotypes from mice engrafted with iPSC-derived neuronal cells.

The presence of large chromosomal abnormalities in COS does affect a vast number of genes, making it challenging to establish clear cause–effect relationships in neurodevelopment. As an exception to this rule, the 2p16.3 microdeletion presents non-recurrent copy number variations that associate only with deletions of *NRXN1* owing its large size. *NRXN1* encodes for a presynaptic cell-adhesion molecule, called neurexin-1, and structural variations in this gene strongly increase risk for both COS and SCZ. Pak et al. [64] recently generated isogenic human ESC lines containing heterozygous conditional *NRXN1* mutations and showed that in neural differentiated cells, heterozygous loss of *NRXN1* specifically impaired the early phase of synaptic transmission. The concept of the ‘tripartite synapse’ highlights the importance of astrocytes as equal partners with neuronal presynaptic and postsynaptic terminals in synaptic processing of information. In light of impaired astroglial function in COS and its strong association with 2p16.3, it would be interesting to investigate astrocytic function in heterozygous *NRXN1* mice [64] as well.

Apart from glial cells, neurons can be likewise transplanted into mice brain. In this regard, Shao et al. [54] recently showed that iPSC-derived cINs from healthy controls and patients with SCZ developed, upon cortical transplantation, into cells closely recapitulating the features of endogenous interneurons: They expressed postsynaptic structures responsive to excitatory glutamatergic host neurons concurrent with presynaptic GABA-releasing structures inhibiting host neurons. Transplanted cINs of either diagnostic group did not differ in terms of cell fate decisions and excitatory synapse formation in vivo. Intriguingly though, a significant cell-autonomous deficit in inhibitory synapse formation was detected for cINs derived from patients’ iPSCs relative to controls. This finding is a significant improvement in insight on excitatory–inhibitory imbalance in SCZ available before though room for improvement remains.

Two recent studies based on human pluripotent stem cells transplanted either cortical progenitors and neurons derived from 2D-monolayer culture [65] or 3D-brain-organoids [60] into adult mice brain. At first, engrafted brain organoids developed corticothalamic and subcerebral projection neurons followed by later intracortical projection neurons. While transplanted brain organoids successfully integrated in the adult cortex, none of the neuronal subtypes traced the laminar organization of the host cortex faithfully. Vascularization of human organoids occurred within two weeks by the host tissue and promoted human astrocyte and oligodendrocyte development concomitant with host microglia invasion. This result opens the possibility to investigate complex cell–cell interactions in vivo, moving beyond the in vitro triculture system described above [64]. To target cortical and subcortical regions, transplanted human neurons also formed long-range connections that underwent, with maturation, substantial structural refinements, indicating that pruning mechanisms were maintained upon transplantation [65]. Hence, previous findings by Sellgren et al. [34] on differences between healthy controls and patients with SCZ in microglia-mediated engulfment of neuronal synapses in vitro could be taken to a more physiological environment. At the same time, axons of human transplanted neurons integrated functionally into existing host circuits and thus could allow modeling of synapse formation and connectivity in vivo [60]. Engrafted brain organoids progressively acquired (i) synchronous network activity, (ii) oscillatory patterns, and (iii) responses to sensory stimuli indicating circuit-level maturation upon grafting [65]. This raises the exciting prospect that transplantation of iPSC-derived brain organoids not only enables us to investigate differences between healthy controls and patients with psychiatric disorders under resting conditions, but also in response to environmental experiences in recipient mice. The brain processes sensory, cognitive, and social information with behavior reflecting circuit physiology that may be altered as the cells constituting the circuit are altered. When the brain mismanages environmental information, risk for psychiatric disorders increases. Thus, circuit-level integration of brain organoids may provide a handle on the study of gene environment interactions in living human neurons under ‘naturalistic’ conditions. SCZ has been dubbed ‘disease of the synapse’ [66] and future transplantation studies of iPSC-derived neural cell from healthy controls and patients with SCZ may provide unprecedented insight into synapse and circuit formation in vivo, and likewise, in response to environmental input. Along this line, such experiments would also be of high interest to psychiatric disorders (e.g., MD), in which gene environment interactions are thought to play a critical role in disease onset and progression.

All in all, iPSC-based disease-modeling of psychiatric disorders has made significant progress over the past years with further improvements in technique and reduction in costs to come in the near future. Though we are still far away to reproduce the complexity of the human brain in vitro or in transplanted animals, current models can offer unpresented insight into the underpinning molecular and cellular mechanisms and how they give rise to the formation of cells, synapses, and circuits from which behavior eventually emerges. Knowledge gained from these studies will not only catalyze new concepts on the classification and etiopathogenesis of psychiatric disorders, but also on the development of new treatments to benefit affected patients and their families in the long term.

## Abbreviations

BD	Bipolar disorder
CA3	cornu ammonis subfield 3
COS	childhood onset of schizophrenia
ESC	embryonic stem cell
DG	dentate gyrus
GABA	γ-aminobutyric
cIN	cortical interneuron
hGPC	human glial progenitor cells
hiMG	human induced microglia
iPSC	induced pluripotent stem cells
NPC	neural progenitor cells
MD	major depression
SCZ	schizophrenia
